# The Study of Practical Anatomy during the Summer Months

**Published:** 1867-05

**Authors:** 


### EDITORIAL AND MISCELLANEOUS

### THE STUDY OF PRACTICAL ANATOMY DURING THE SUMMER MONTHS.

Yearly observation has confirmed us in the opinion advanced several years ago, that in warm weather dissections can be more conveniently, pleasantly, and profitably made than in winter. In this climate, subjects not prepared are of but little use in summer or winter. And even in northern latitudes, bodies kept in rooms sufficiently warm to make the student at all comfortable, will very soon become offensive and petrid. The modern mode of preserving bodies, then, is decidedly preferable for their use any season of the year. When properly injected subjects can be kept for an indefinite length of time, by keeping them submerged in fluid of some suitable kind, so as to prevent them from becoming dry and hard. We have known them kept in this way for four years, and dissected after the lapse of that time. The dissecting class in the Atlanta Medical College used subjects in the session after the close of the war, which were prepared in 1861. Dissections are now going on in the College, by students coming on to prosecute the study of Practical Anatomy, in advance of the opening of the lectures in May, very satisfactory to the class engaged. The great advantage of dissecting in warm weather, particularly when the dissecting-room is even less offensive than those kept for winter dissections, is readily perceptible to students who, with benumbed fingers and shivering limbs, disseeted during last winter. Dissecting classes will be accommodated during the whole summer, throughout the course of lectures, as usual, in the use of material not at all putrid or offensive.

These facts having been verified for ten years, to the entire satisfaction of those taking the trouble to visit this Institution during its sessions, the great objection so vigorously urged against summer medical teaching heretofore, falls to the ground, and the result is, that the patronage of the College has steadily increased. Of course, the great convulsion to which the country has recently been subjected, has brought prosperous enterprises of all kinds to commence as it were, *de novovo;* but we observe, since the war, the same tendency to yearly increase of the number of students in attendance, as before. The indications, at present, lead us to expect double the number the ensuing session of that in attendance last year. That branch of science—Practical Anatomy—which it was thought by many would have to be virtually ignored by Summer Schools, will be found a leading inducement to the patronage of such Institutions. With a well lighted and ventilated dissecting-room, and well prepared material, we have no hesitancy in saying, that the warm seasons give great advantages in the study of Practical anatomy.

